# Agreement of Magnetic Resonance Imaging With Computed Tomography in the Assessment for Acute Skull Fractures in a Canine and Feline Cadaver Model

**DOI:** 10.3389/fvets.2021.603775

**Published:** 2021-04-22

**Authors:** Silke Hecht, Kimberly M. Anderson, Aude Castel, John F. Griffin, Adrien-Maxence Hespel, Nathan Nelson, Xiaocun Sun

**Affiliations:** ^1^Department of Small Animal Clinical Sciences, University of Tennessee, Knoxville, TN, United States; ^2^Department of Large Animal Clinical Sciences, College of Veterinary Medicine and Biomedical Sciences, Texas A&M University, College Station, TX, United States; ^3^Department of Molecular and Biomedical Sciences, North Carolina State University, Raleigh, NC, United States; ^4^Office of Information Technology, University of Tennessee, Knoxville, TN, United States

**Keywords:** MRI, CT, trauma, head, CNS, dog, cat

## Abstract

Computed tomography (CT) is the imaging modality of choice to evaluate patients with acute head trauma. However, magnetic resonance imaging (MRI) may be chosen in select cases. The objectives of this study were to evaluate the agreement of MRI with CT in the assessment for presence or absence of acute skull fractures in a canine and feline cadaver model, compare seven different MRI sequences (T1-W, T2-W, T2-FLAIR, PD-W, T2^*^-W, “SPACE” and “VIBE”), and determine agreement of four different MRI readers with CT data. Pre- and post-trauma CT and MRI studies were performed on 10 canine and 10 feline cadaver heads. Agreement of MRI with CT as to presence or absence of a fracture was determined for 26 individual osseous structures and four anatomic regions (cranium, face, skull base, temporomandibular joint). Overall, there was 93.5% agreement in assessing a fracture as present or absent between MRI and CT, with a significant difference between the pre and post trauma studies (99.4 vs. 87.6%; *p* < 0.0001; OR 0.042; 95% CI 0.034–0.052). There was no significant difference between dogs and cats. The agreement for the different MRI sequences with CT ranged from 92.6% (T2^*^-W) to 94.4% (PD-W). There was higher agreement of MRI with CT in the evaluation for fractures of the face than other anatomic regions. Agreement with CT for individual MRI readers ranged from 92.6 to 94.7%. A PD-W sequence should be added to the MR protocol when evaluating the small animal head trauma patient.

## Introduction

Head trauma in dogs and cats is associated with a high morbidity and mortality. Possible causes include road traffic accidents, falls, injuries caused by other animals (e.g., kicks or bites), and human inflicted trauma including ballistic injuries and abuse (1–8).

Advanced imaging of the head may be performed for assessment of the type and extent of intracranial injuries, therapeutic planning, and prognostication (1, 4, 7, 8). Computed tomography (CT) is generally considered the modality of choice to evaluate patients with acute head trauma (9). It is quick, does not require general anesthesia, and is highly accurate in the diagnosis of conditions which may impact clinical management such as fractures, intracranial hemorrhage, brain swelling and brain herniation (2, 10–17). In people, magnetic resonance imaging (MRI) is indicated in patients with acute traumatic brain injury when CT fails to explain the neurologic findings, and it is the preferred imaging modality for the evaluation of subacute and chronic brain trauma (10, 15, 16, 18). While MRI and CT have similar sensitivity in the detection of acute epidural and subdural hematomas (19, 20), MRI is superior in the detection of non-hemorrhagic lesions, brainstem injuries and subarachnoid hemorrhage (20–22). However, MRI is limited in its ability to evaluate cortical bone which is characterized by low proton density and very short T2 relaxation time (23). Another disadvantage of MRI compared to CT is the need for general anesthesia.

Performing both CT and MRI in a small animal trauma patient to optimize diagnostic information for both soft tissues and bone is often cost prohibitive and requires longer anesthetic time in potentially unstable patients. CT is increasingly available in small animal practice and will likely be given preference in many instances. However, MRI may be chosen if CT is unavailable or if improved soft tissue imaging is desired. Advanced MRI techniques that are being developed for improved cortical bone imaging in human trauma patients (23–25) may not be available for veterinary MRI systems or may be cost prohibitive.

The objectives of this study were to (1) evaluate the overall agreement of MRI with CT in the assessment for presence or absence of acute skull fractures in a canine and feline cadaver model, (2) identify differences in fracture detection between dogs and cats, (3) determine the agreement of seven different standard MRI sequences with CT as to the presence or absence of fractures, (4) determine the agreement of MRI with CT as to the presence or absence of fractures for different anatomic regions of the head, and (5) determine agreement of four different MRI readers with CT data. Hypotheses were that (1) MRI would have high agreement with CT in assessing presence or absence of acute skull fractures, (2) there would not be a significant difference between dogs and cats, (3) sequences with a short time of echo (TE) (T1-weighting (T1-W) and proton density weighting (PD-W)) and thinner slice thickness (T2-weighted turbo spin echo with Variable Flip Angle (“Sampling Perfection with Application optimized Contrasts using different flip angle Evolution”, “SPACE”) and T1-W Volume Interpolated gradient recalled echo (GRE) images with fat saturation (“Volume Interpolated Breathhold Examination”, “VIBE”) would have higher agreement with CT compared to other sequences (T2-W SE, T2^*^-W GRE and T2-FLAIR), (4) there would be no difference in the ability to determine presence or absence of fractures between different anatomic areas of the skull, and that (5) there would be no difference between four observers.

## Materials and Methods

This study is a prospective, experimental, diagnostic accuracy, methods comparison cadaver study. A STARD (“Standards for Reporting Diagnostic accuracy studies”) checklist was followed during manuscript preparation.

### Animals

Approval by the Institutional Animal Care and Use Committee was not required for this cadaveric study. Ten canine and 10 feline cadaver heads were used for the project. All animals were humanely euthanized for purposes other than the current study and were provided by local shelters. Gross (visual) evidence of pre-existing head trauma and deformities of the head were considered exclusion criteria. Two different methods for inducing skull injuries were employed to mimic blunt force trauma commonly seen in companion animals. Half each of the canine and feline heads were dropped from a height of about 15 feet to mimic a fall, and the other half were hit with an object (hammer or door) in a way to mimic the impact from a kick or bite wound. Each head from the dog and cat cohort was randomly assigned to one of the trauma methods.

### Imaging Protocol

CT and MRI were performed twice on each cadaver head, once before and once after the induction of skull trauma.

CT was performed using a 40-slice helical CT scanner (Philips Brilliance-40™, Philips International B.V., Amsterdam, Netherlands). Images were acquired in a transverse plane using a helical acquisition. The slice thickness was 0.9 mm with a pitch of 0.5. The tube rotation was 1.1s with a milliamperage (mA) range of 188–237 and peak kilovoltage (kVp) of 120. The matrix was set at 512 × 512. The field of view ranged from 94 to 150 mm. Images were reconstructed using a bone algorithm. A bone window was used for evaluation (preset window center 600 HU, window width 2600 HU). The window display was not restricted allowing observers to adjust the display. Multiplanar or three-dimensional reformatting was not performed. Contrast medium was not administered.

MRI was performed using a 1.5 Tesla superconducting MRI system (MAGNETOM Espree™, Siemens Medical Solutions, Malvern, PA) equipped with a head coil. Seven MRI sequences with transverse slice orientation were acquired for subsequent image evaluation: T2-weighted turbo spin echo (TSE) (T2-W), T1-weighted SE (T1-W), T2-weighted fluid attenuated inversion recovery (T2-FLAIR), proton density weighted TSE (PD-W), T2^*^-weighted gradient recalled echo (GRE) images (T2^*^-W), transverse T2-weighted TSE with Variable Flip Angle (“Sampling Perfection with Application optimized Contrasts using different flip angle Evolution”, “SPACE”) and T1-weighted Volume Interpolated GRE images with fat saturation (“Volume Interpolated Breathhold Examination”, “VIBE”). Contrast medium was not administered. Acquisition parameters are detailed in Table 1.

**Table 1:** Acquisition parameters of MRI sequences.

**Sequence**	**Sequence type**	**TR (ms)**	**TE (ms)**	**TI (ms)**	**Flip angle (^**°**^)**	**NEX**	**Slice thickness (mm)**	**Interslice gap (%)**	**Field of view (cm)**	**Acquisition matrix**
T2-W TSE	2D	3,820–4,880	102–109	N/A	90°	1–2	3–4	10	12–16	256 × 192
T1-W SE	2D	330–437	12	N/A	90°	1–2	3–4	10	12–16	256 × 192
T2-FLAIR	2D	7,000–8,980	75–77	2,215–2,496	90°	1	3–4	10	12–16	256 × 192
PD-W TSE	2D	2,000–2,300	13–14	N/A	90°	1–2	3–4	10	12–16	256 × 192
T2*-W GRE	2D	850–1,220	26	N/A	20°	1	3–4	10	12–16	256 × 192
“SPACE”	3D	1,300	123	N/A	variable	2	1–1.2	N/A	12–16	256 × 192
“VIBE”	3D	5.5–5.55	2.39	N/A	10°	1–2	1–1.2	N/A	12–16	256 × 192

TSE, turbo spin echo; SE, spin echo; GRE, gradient recalled echo; T2-W, T2-weighted; T1-W, T1-weighted; T2-FLAIR, T2-weighted fluid attenuated inversion recovery; PD-W, proton density-weighted; T2^*^-W, T2^*^-weighted; “SPACE”, “sampling perfection with application optimized contrasts using different flip angle evolution”; “VIBE”, “volume interpolated breathhold examination”; TR, time of repetition; TE, time of echo; TI, time of inversion; NEX, number of excitations; ms, milliseconds; mm, millimeter; cm, centimeter.

### Image Evaluation

CT was considered the gold standard for the purpose of this study. The pre and post trauma CT studies were independently evaluated by the two PIs (KMA, an ACVIM board-certified veterinary neurologist, and SH, an ACVR and ECVDI board-certified veterinary radiologist) not blinded to trauma status, and a consensus was reached in case of disagreement. Pre-trauma CT was performed to identify any cases with evidence of pre-existing acute traumatic lesions. Twenty six osseous structures of the head were evaluated individually for the presence of fractures (individual basisphenoid bone, presphenoid bone, ethmoid bone/cribriform plate and hard palate; and paired occipital bone, parietal bone, frontal bone, temporal bone “cranium”, temporal bone “temporomandibular joint (TMJ; mandibular fossa of the temporal bone)”, incisive bone, nasal bone, maxilla, zygomatic arch, mandible “ramus and body”, and mandible “TMJ; condyle of the mandible”). No qualitative information about the fractures was recorded.

Subsequently, all MRI sequences (seven pre- trauma and seven post-trauma for each of the 20 cadaver heads) were anonymized and randomized. Evaluation of the individual sequences was independently performed by three ACVR board-certified veterinary radiologists (JFG, AMH, NN) and one ACVIM board-certified veterinary neurologist (AC) from three different institutions and blinded to trauma status and results of the CT examinations. Both pre trauma MRI studies (negative controls) and post trauma studies were provided to the readers for independent evaluation. Images were provided to investigators in DICOM format and were evaluated on a DICOM viewing software of their choice. For each individual sequence readers were asked to evaluate each individual osseous structure and mark fracture “present” or “absent” on an Excel data sheet (see Supplementary File 1). Qualitative fracture information was not collected. Agreement of the seven MRI sequences with CT in fracture assessment was determined for the 26 osseous structures listed above. Additionally, these osseous structures were grouped by anatomic region as follows: Bones forming the cranium (occipital, parietal, frontal, and temporal bones); bones forming the face (incisive, nasal, maxilla, and zygomatic arch); bones forming the temporomandibular joints (TMJ; mandibular fossa of the temporal bone and condyle of the mandible); and bones of the skull base (basisphenoid and presphenoid bones). The MRI evaluation results for each reader were compared to CT as “match” or “no match” for fracture status (present or absent) of individual bones.

### Statistical Analysis

The statistical analysis was performed by a university employed statistician (XS). The effects of trauma status (pre and post trauma), species (dog and cat), MRI sequence, fracture location, and reader on the evaluation status (agreement between MRI and CT in fracture identification as match or no match) were analyzed using generalized linear mixed model analysis with the evaluation status as the binary response variable and individual bones as the random effect. Odds ratios were calculated for all independent variables. Qualitative data are presented as count numbers and percentages. The chance of fracture detection for the qualitative independent variables was calculated as the probability that an observation will be correctly classified. Statistical significance was identified at the level of 0.05. *Post-hoc* power analysis was conducted to confirm that the sample size was large enough to ensure that a two-sided test with α = 0.05 would yield at least 80% power to detect the effects of the independent variables. Analyses were conducted in SAS 9.4 TS1M6 for Windows 64 × (SAS institute Inc., Cary, NC).

## Results

### Animals

The sex, breed, and weight of individual animals were not available as in several cases only the head but not the entire cadaver was provided to the investigators. All dogs were medium to large breeds with mesaticephalic or dolichocephalic head conformation, and all cats had head conformation consistent with domestic cats. None of the cadavers had visual evidence of pre-existing head trauma or skull deformity, and no cases were excluded from the study.

### Fractures

Ninety four fractures were induced in 19 cadaver skulls. One feline skull was traumatized with no fractures found on CT; this case remained assigned to the post trauma group. At least one fracture was induced in each of the other skulls. The median number of fractures in each skull was 3.5 (range, 0–17). Fractures involved the occipital bone (*n* = 6), parietal bone (*n* = 8), frontal bone (*n* = 18), temporal bone (cranium) (*n* = 7), temporal bone (TMJ—mandibular fossa) (*n* = 8), incisive bone (*n* = 3), nasal bone (*n* = 2), maxilla (*n* = 8), zygomatic arch (*n* = 6), basisphenoid bone (*n* = 1), presphenoid bone (*n* = 5), ethmoid bone/cribriform plate (*n* = 4), hard palate (*n* = 3), mandible (ramus and body) (*n* = 10), and mandible (TMJ—condyle of the mandible) (*n* = 5).

#### Dogs

Forty nine fractures were induced in 10 cadaver skulls. The median number of fractures in each skull was 3.5 (range, 1–17). Fractures involved the occipital bone (*n* = 4), parietal bone (*n* = 2), frontal bone (*n* = 11), temporal bone (cranium) (*n* = 2), temporal bone (TMJ—mandibular fossa) (*n* = 4), incisive bone (*n* = 3), nasal bone (*n* = 2), maxilla (*n* = 5), zygomatic arch (*n* = 4), ethmoid bone/cribriform plate (*n* = 2), hard palate (*n* = 3), mandible (ramus and body) (*n* = 5), and mandible (TMJ—condyle of the mandible) (*n* = 2).

#### Cats

Forty five fractures were induced in nine cadaver skulls. The median number of fractures in each skull was 3.5 (range, 0–11). Fractures involved the occipital bone (*n* = 2), parietal bone (*n* = 6), frontal bone (*n* = 7, temporal bone (cranium) (*n* = 5), temporal bone (TMJ—mandibular fossa) (*n* = 4), nasal bone (*n* = 2), maxilla (*n* = 3), zygomatic arch (*n* = 2), basisphenoid bone (*n* = 1), presphenoid bone (*n* = 5), ethmoid bone/cribriform plate (*n* = 2), mandible (ramus and body) (*n* = 5), and mandible (TMJ—condyle of the mandible) (*n* = 3).

Basisphenoid and presphenoid fractures were only seen in cats, while fractures of the nasal bones, incisive bones and hard palate were only seen in dogs.

### Data Evaluation

A table with the evaluation data and graphic data plots are provided with the supplementary documents (Supplementary Files 2,3).

Twenty nine thousand one hundred twenty data points were available for statistical evaluation (20 cadaver heads, 2 MRI studies each, 7 MRI sequences per study, 26 individual osseous structures per skull, and 4 readers).

Overall, there was 93.5% agreement between MRI and CT in fracture assessment. There was a significant difference in agreement of MRI with CT between the pre and post trauma studies (99.4 vs. 87.6%; *p* < 0.0001; OR 0.042; 95% CI 0.034–0.052). On pre-trauma studies, MRI evaluation matched CT in 14,468/14,560 possible data points, while post trauma studies matched CT in 12,760/14,560 data points.

There was no significant difference in agreement between MRI and CT between dogs and cats (94.1 vs. 92.9%; *p* = 0.5175; 95% CI 0.663–2.260).

**Figure 1:**
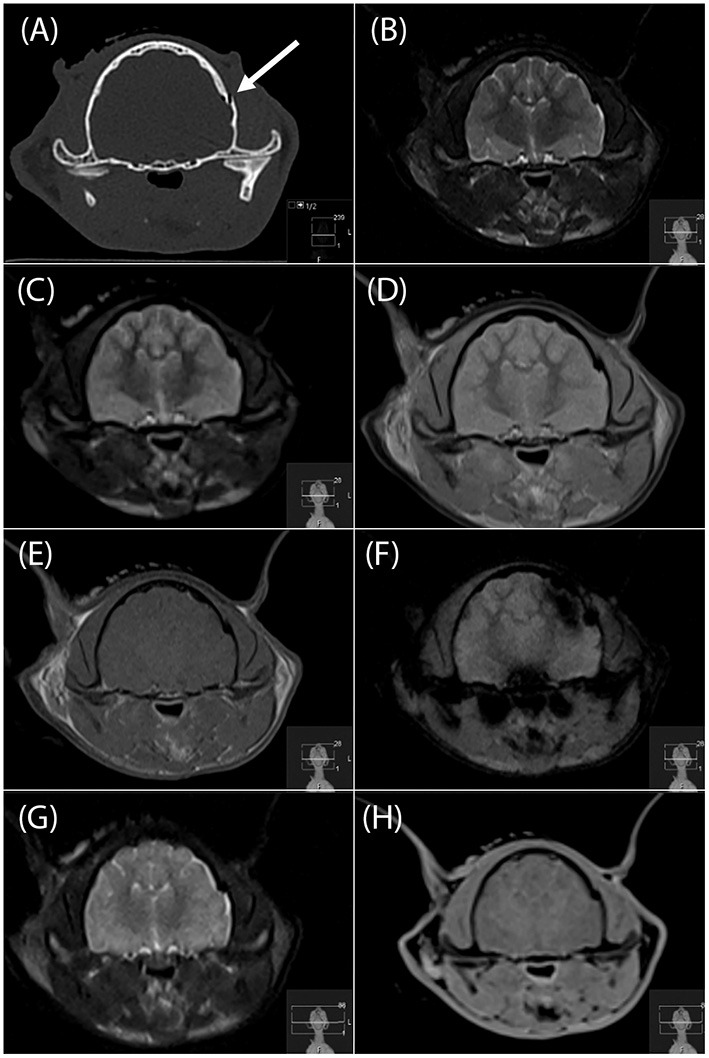
Transverse CT image **(A)**, and transverse MR images (**B**, T2-W; **C**, T2-FLAIR; **D**, PD-W; **E**, T1-W; **F**, T2^*^-W; **G**, “SPACE” and H, “VIBE”) of a left temporal bone fracture in a cat (arrow in a). This fracture was identified by all four readers on the PD-W sequence **(D)**, 3 readers on the T1-W sequence and “SPACE” sequences **(E,G)**, two readers on the T2-W and “VIBE” sequences **(B,H)** and no reader on the T2^*^-W sequence **(F)**. FLAIR, fluid attenuated inversion recovery; PD, proton density; “SPACE”, sampling perfection with application optimized contrasts using different flip angle evolution; “VIBE”, volume interpolated breathhold examination.

The agreement for the seven different MRI sequences with CT ranged from 92.6% (T2^*^-W GRE) to 94.6% (PD-W). The T2^*^-W GRE sequence had significantly lower odds of accurate fracture assessment compared to PD and “VIBE” sequences, and the PD-W sequence had significantly higher odds of accurate fracture assessment compared to T2-W sequences (*p* = 0.0360; Table 2 and Figure 1).

**Table 2:** Agreement of different MRI sequences with CT as the gold standard in the assessment for skull fractures.

**Sequence**	**Agreement with CT**	**Comparison to FLAIR (OR; 95% CI)**	Comparison to T2* GRE (OR; 95% CI)	**Comparison to PD (OR; 95% CI)**	**Comparison to “SPACE” (OR; 95% CI)**	**Comparison to T1 (OR; 95% CI)**	**Comparison to T2 (OR; 95% CI)**	**Comparison to “VIBE” (OR; 95% CI)**
T2-FLAIR	93.4%		1.126 (0.948–1.337)	0.838 (0.698–1.005)	0.996 (0.835–1.188)	0.948 (0.794–1.132)	1.053 (0.884–1.253)	0.912 (0.763–1.091)
T2* GRE	92.6%			**0.744 (0.622**–0.889)*	0.884 (0.745–1.050)	0.842 (0.707–1.001)	0.935 (0.789–1.108)	**0.810 (0.680**–0.965)*
PD	94.4%				1.189 (0.991–1.428)	1.132 (0.941–1.361)	**1.257 (1.049**–1.506)*	1.089 (0.904–1.312)
“SPACE”	93.4%					0.952 (0.797–1.137)	1.057 (0.888–1.258)	0.916 (0.766–1.096)
T1	93.7%						1.110 (0.931–1.324)	0.962 (0.803–1.153)
T2	93.1%							0.867 (0.726–1.035)
“VIBE”	93.9%							

CT, computed tomography; T2-FLAIR, T2-W fluid attenuated inversion recovery; T2^*^ GRE, T2^*^-W gradient recalled echo; PD, proton density; “SPACE”, sampling perfection with application optimized contrasts using different flip angle evolution; “VIBE”, volume interpolated breathhold examination; OR, odds ratio; CI, confidence interval.

^*^Statistically significant difference (p = 0.0360).

The agreement of MRI with CT was 92.7% for the temporomandibular joint, 92.8% for the cranium, 93.4% for the skull base, and 95.1% for the face. The odds of accurate fracture assessment were significantly higher for facial fractures compared to the other three anatomic regions (*p* < 0.0001; Table 3).

**Table 3:** Agreement of MRI with CT in the assessment for fractures at various anatomic locations.

**Anatomic region**	**Agreement with CT**	**Comparison to cranium (OR; 95% CI)**	**Comparison to Skull base (OR; 95% CI)**	**Comparison to face (OR; 95% CI)**	**Comparison to TMJ (OR; 95% CI)**
Cranium	92.8%		0.910 (0.754–1.097)	**0.655 (0.577**–0.743)^*^	1.007 (0.898–1.129)
Skull base	93.4%			**0.720 (0.592**–0.874)^*^	1.107 (0.918–1.335)
Face	95.1%				**1.538 (1.355**–1.745)^*^
TMJ	92.7%				

Cranium: Occipital, parietal, frontal, and temporal bones.

Skull base: Basisphenoid and presphenoid bones.

Face: Incisive bones, nasal bones, maxillae, and zygomatic arches.

Temporomandibular joints (TMJ: Mandibular fossa of the temporal bone and condyle of the mandible).

CT = Computed Tomography; TMJ = temporomandibular joint; OR = odds ratio; CI = confidence interval.

* *Statistically significant difference (p < 0.0001)*.

**Figure 2:**
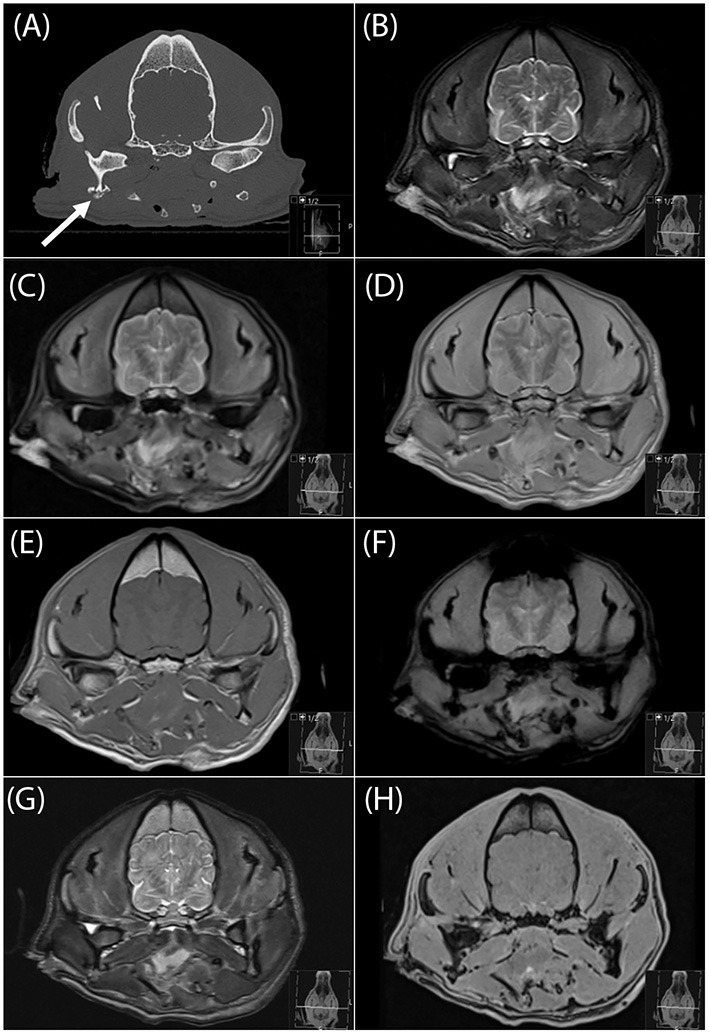
Transverse CT image **(A)**, and transverse MR images (**B**, T2-W; **C**, FLAIR; **D**, PD-W; **E**, T1-W; **F**, T2*-W; **G**, “SPACE” and **H**, “VIBE”) of a focal comminuted fracture of the angular process of the right mandible (arrow in a). This was the only fracture in this cadaver skull and was missed by all four readers on all sequences. FLAIR, fluid attenuated inversion recovery; PD, proton density; “SPACE,” sampling perfection with application optimized contrasts using different flip angle evolution; “VIBE,” volume interpolated breathhold examination.

When considering the head as a whole, at least one fracture was identified on MRI on at least one sequence by all readers in 17/19 cases, and at least one fracture was identified on MRI on at least one sequence by at least one reader in 18/19 cases. The single case in which MRI (all readers and all sequences) failed to correctly identify the single fracture present had a focal comminution of the angular process of the right mandible (Figure 2). Additional fractures missed included fractures of the occipital bone (*n* = 2), frontal bone (*n* = 1), temporal bone (TMJ—mandibular fossa) (*n* = 1), incisive bone (*n* = 1), maxilla (*n* = 1), hard palate (*n* = 1), mandible (ramus and body) (*n* = 5), and mandible (TMJ—condyle of the mandible) (*n* = 4). At least one addition fracture present in each of these cases was correctly identified. In the pre-trauma assessments, all osseous structures were correctly identified as normal in four cases. Sporadic (two or less per reader) false positive diagnoses were made on 15 skulls, with false positive diagnoses combined for all bones, readers and sequences ranging from 1 to 12 (out of 728 data points per head total). A chronic anomaly of the right frontal sinus in one feline skull was mistaken for an acute fracture by all readers on two to four sequences.

Four blinded readers were utilized in this study, three board certified radiologists and one board certified neurologist. Fracture assessment by individual readers ranged from 92.6 to 94.7% with one reader having significantly higher agreement with CT than the other three readers (*p* < 0.0001).

## Discussion

The results of this study confirmed our hypotheses that (1) MRI would have high agreement (93.5%) with CT in the assessment for presence or absence of acute skull fractures in a canine and feline cadaver model, and (2) that there would be no statistically difference between dogs and cats. While CT has traditionally been the modality of choice for the initial assessment of acute head trauma and traumatic brain injury in people, MRI is preferred in many cases of subacute and chronic head trauma and for improved visualization, especially of brain parenchymal non-hemorrhagic lesions (15, 26–29). Several studies have compared MRI to CT in human head trauma patients. In one study of 30 children with head trauma, MRI missed skull fractures in 5 of 13 patients (30). In another study in young children, MRI missed skull fractures in 6 out of 81 patients (31). Increasing concerns with radiation exposure in CT, along with the recognition of excellent soft tissue capabilities of MRI, have prompted several studies aimed at overcoming the limitations of MRI with regards to bone imaging. A study investigating Zero TE Skull MRI found similar diagnostic quality of MR images compared with CT in the detection of skull fractures in 13/13 patients (24). In another study, black bone MRI with 3D reconstruction found 19/20 skull fractures in children (25). Similar to people, CT is typically the first line imaging test in small animal patients presented with traumatic head injury (4). However, the improved soft tissue imaging capabilities of MRI compared to CT provide not only diagnostic but also prognostic information, making it a desirable tool in the assessment of the small animal head trauma patient (1, 7, 8). Unfortunately, several of the new imaging sequences optimized for bone imaging are not available for MRI systems used in veterinary hospitals or are cost prohibitive at this point. This study was therefore focused on MRI sequences available on a standard 1.5T MRI system, hoping to provide useful clinical information for veterinary teaching hospitals and private practices alike. When considering the head as a whole, at least one fracture was identified on MRI on at least one sequence by at least one reader in 18/19 cases. While this is a high number, several missed lesions in cases with multiple fractures underlines that CT may still be needed in some patients if a skull fracture is of concern but not identified on MRI.

The third hypothesis was that sequences with a short TE (T1-W and PD-W) and thinner slice thickness would have higher agreement with CT compared to other sequences. This was confirmed. The PD-W sequence had the highest agreement with CT (94.4%) in fracture assessment. This is not surprising as it provides excellent contrast between dense cortical bone and bone marrow and for this reason is considered a mainstay in human and veterinary orthopedic imaging (32, 33). The sequence with the second highest agreement in this study was the “VIBE” sequence, a thin section 3D T1-weighted GRE sequence with fat suppression originally developed for abdominal breath hold MRI in people (34) but also utilized for brain imaging (35, 36). Advantages of this technique for bone evaluation include a thin slice thickness combined with T1-weighting. An additional advantage in a clinical setting is the multiplanar reconstruction capability of the 3D data set to allow for fracture evaluation in different planes. This potential additional advantage of this sequence was not explored in this project and could be investigated in a follow-up project using the same dataset. The regular T1-W SE sequence performed similarly well as the “VIBE” sequence in this study (93.7 vs. 93.9% agreement with CT). The second thin section sequence tested in this study (“SPACE,” a three-dimensional T2-weighted turbo spin echo sequence) was also slightly better compared to the regular T2-W SE sequence obtained with thicker slices (93.4 vs. 93.1%). Despite these differences between sequences it is important to note that the agreement of all individual MRI sequences with CT was high (above 90%), and that the only significant differences between sequences were between the T2^*^-W and PD-W, the T2^*^-W and “VIBE,” and the PD-W and T2-W sequence, respectively. The T2^*^-W GRE sequence had least agreement with CT (92.6%) which is unsurprising. This gradient echo sequence uses a low flip angle (20°), resulting in a low echo amplitude and an inherently lower signal-to-noise ratio compared to SE sequences (37). Additionally, subcutaneous and intracranial susceptibility artifacts related to trauma and postmortem gas accumulation commonly resulted in susceptibility artifacts, limiting the assessment of adjacent bony structures (see study limitations below).

The fourth hypothesis was that there would be no difference in the ability to determine presence or absence of fractures between different anatomic areas of the skull. This hypothesis was rejected. MRI was significantly better in assessing for fractures affecting the face than other areas including the cranium. While this information is interesting, it will likely not result in a change in clinical practice. The overarching goal of this and similar studies is to identify instances where MRI can be used in lieu of CT to provide adequate concurrent assessment of traumatic brain injuries and associated fractures of the cranial vault. Despite the good diagnostic performance of MRI in facial fracture assessment in this study, CT will likely remain the gold standard for imaging of animals with maxillofacial trauma, especially when planning surgical interventions (38, 39). MRI had least agreement with CT in the detection of TMJ fractures. Even though both CT and MRI may be used for evaluation of patients with TMJ diseases, CT is typically given preference for evaluation of the osseous structures (40). The agreement between MRI and CT for fracture assessment of the cranium and skull base was high and may have even been improved with a different study design. Investigators were presented with individual MRI sequences rather than the entire set of sequences to allow comparison of the diagnostic yield of different sequences. This is unrealistic from a clinical perspective where several sequences are typically acquired and used in conjunction for clinical decision making.

The fifth and last hypothesis that there would be no difference between four observers was also rejected. While all readers had high agreement with CT, one reader had a significantly higher agreement than the others. Unfortunately, there were not enough readers to evaluate effects of years of experience or type of board certification.

There are several limitations to this study. Despite best efforts to mimic naturally occurring head trauma, skull fractures were induced artificially and may not be representative of naturally occurring fractures, or the type and severity of these artificially induced fractures may be incompatible with patient survival to diagnostic imaging. Prior to this study, investigators attempted multiple additional ways to induce skull fractures and were often unsuccessful especially in large breed dogs. In case of multiple, extensive, and comminuted fractures, identification of which exact bones were affected was sometimes challenging, even on CT which was used as the gold standard. We attempted to minimize this problem by having two investigators read the CT studies independently and then reach an agreement by consensus. For fractures following suture lines that could not be unequivocally attributed to a specific bone, readers were given credit when they assigned the fracture to either of the two bones bordering the suture.

**Figure 3:**
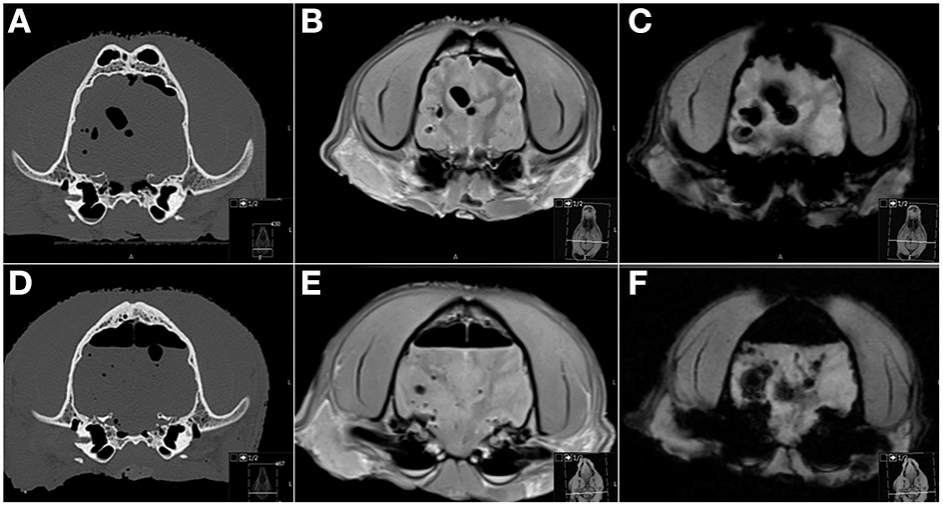
Transverse CT **(A,D)**, PD-W **(B,E)** and T2*-W **(C,F)** MR images before **(A–C)** and after **(D–F)** induction of skull trauma. **(A–C)** Even on the “pre trauma” images a moderate amount of gas is associated with the cranial vault and brain parenchyma, attributed to prior cadaver decapitation and postmortem status and resulting in marked susceptibility artifacts especially on the T2*-W image **(C)**. **(D–F)** The post trauma images were acquired almost 6 h later and are characterized by an increase in intra-cranial gas accumulations and associated susceptibility artifacts. PD, proton density.

Accumulation of gas within the cranial vault and in some cases subcutaneous tissues was a major problem in some cases, was worse for cadavers that had been decapitated prior to imaging, and was progressive over time. As the cadaver studies had to be scheduled to accommodate the clinical schedule, there was often a several hour time lapse between the initial (pre trauma) and the post trauma MRI study. Progressive gas accumulation over time and associated susceptibility artifacts on MRI made the evaluation progressively difficult (Figure 3).

**Figure 4:**
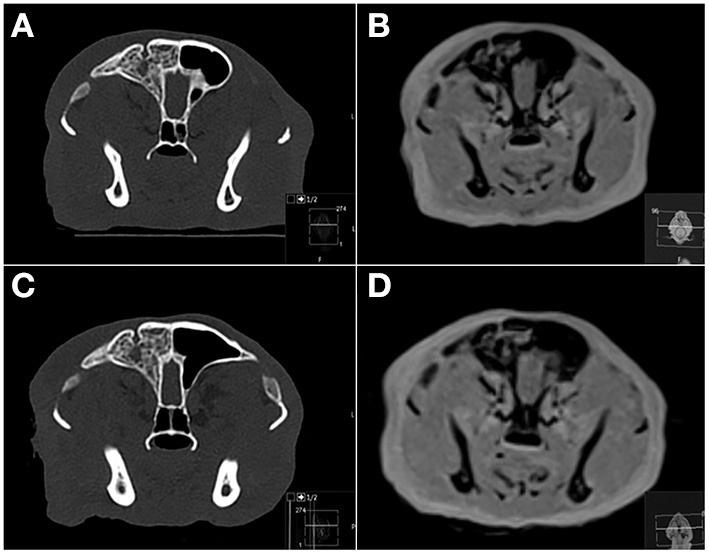
Transverse CT **(A,C)** and PD-W MRI **(B,D)** images of a feline skull before **(A,B)** and after **(C,D)** induction of head trauma. The right frontal sinus is filled with material of heterogeneous bone attenuation, and there is a focal smoothly marginated defect bordered by an osseous rim associated with the dorsal right frontal bone. Based on CT these changes were considered chronic and most consistent with previous trauma or a congenital anomaly. On MRI this lesion was mistaken for an acute fracture by all readers on 2–4 sequences. PD, proton density.

Pre-existing lesions were a minor problem in this study as the cadaver heads were visually prescreened prior to being enrolled in the study. However, a chronic anomaly of the right frontal sinus in one feline skull (Figure 4), believed to be likely congenital or related to prior trauma based on CT, was mistaken for an acute fracture by all readers on two to four sequences. This skull was not excluded from the study as the goal was to determine agreement of MRI with CT in acute skull trauma, and chronic or congenital osseous anomalies may pose a potential imaging pitfall in head trauma imaging of clinical patients. Traumatic soft tissue changes could not be evaluated in this postmortem model. In a live patient, lack of swelling and intensity changes of soft tissue overlying this abnormality may have aided in differentiation between a chronic lesion and acute trauma.

Finally, reader factors may have affected the outcome of this study. It is likely that an investigator paid closer attention to the remainder of the skull once a fracture was identified. However, this is likely the case with interpretation of clinical trauma head MRI studies as well and is not considered a significant study limitation. Even though all specimen and MRI sequences were randomized, a certain “reader memory” for very distinctive features of a given head cannot be excluded. For example, if a skull had a very obvious mandibular fracture and a less obvious skull base fracture, a reader might have recognized the particular mandibular fracture when evaluating a new sequence and may have specifically looked for additional lesions he/she remembered being there. Even though this is a possible study limitation, it is considered unlikely that this played a major role considering the sheer number of individual MRI sequences evaluated by each reader (280). Readers were neither given a timeline to read the studies nor asked to limit the number of sequences interpreted on a given day. One reader reported that he felt very fatigued at the end of the data evaluation and stated that this may have negatively affected his performance. It is likely that all readers experienced some degree of fatigue. However, considering the overall good to excellent agreement between MRI interpretation and CT, this does not appear to have played a major role in the study outcome.

Future studies could include a comparison of CT and MRI examinations in small animal patients with naturally occurring head trauma and taking into account traumatic soft tissue lesions in addition to fractures, a study with an increased number of readers of various experience levels and backgrounds to further evaluate interobserver variability, and studies investigating newer MRI sequences optimized for bone imaging as they become available for MRI systems in veterinary practice.

## Concluding Remarks

MRI has high agreement with CT in skull fracture assessment in a canine and feline cadaver model and may be an acceptable alternate modality if CT is unavailable or if the main indication for imaging is soft tissue evaluation. Fractures were missed in several instances, underlining the need for follow-up CT imaging if there is clinical concern for fractures but MRI is negative. Of the sequences evaluated, the PD-W sequence had highest agreement with CT. As it may not be considered a routine sequence for brain MR imaging at some institutions, addition of this sequence to the protocol should be considered when imaging head trauma patients.

## Data Availability Statement

The original contributions presented in the study are included in the article/Supplementary Material, further inquiries can be directed to the corresponding author/s.

## Ethics Statement

Ethical review and approval was not required for the animal study because the study was performed on cadavers.

## Author Contributions

SH and KA devised the study and prepared the manuscript. AC, JG, A-MH, and NN performed MRI evaluations. SH, KA, and XS performed the data analysis and interpretation. AC, JG, A-MH, NN, and XS revised the manuscript. Final approval of the completed article done by all authors.

## Conflict of Interest

The authors declare that the research was conducted in the absence of any commercial or financial relationships that could be construed as a potential conflict of interest.
