# Supplementary material for: Agreement of Magnetic Resonance Imaging With Computed Tomography in the Assessment for Acute Skull Fractures in a Canine and Feline Cadaver Model
Source: Front Vet Sci. 2021 Apr 22;8:603775. doi: 10.3389/fvets.2021.603775 (PMC8100023; doi:10.3389/fvets.2021.603775)

MRI vs. CT in Head Trauma – Supplement 3

Plot comparisons for MRI agreement with CT (“match” vs. “no match”)

A) Plot of match vs. no match assessments for trauma status (pre = pre trauma; post = post trauma)

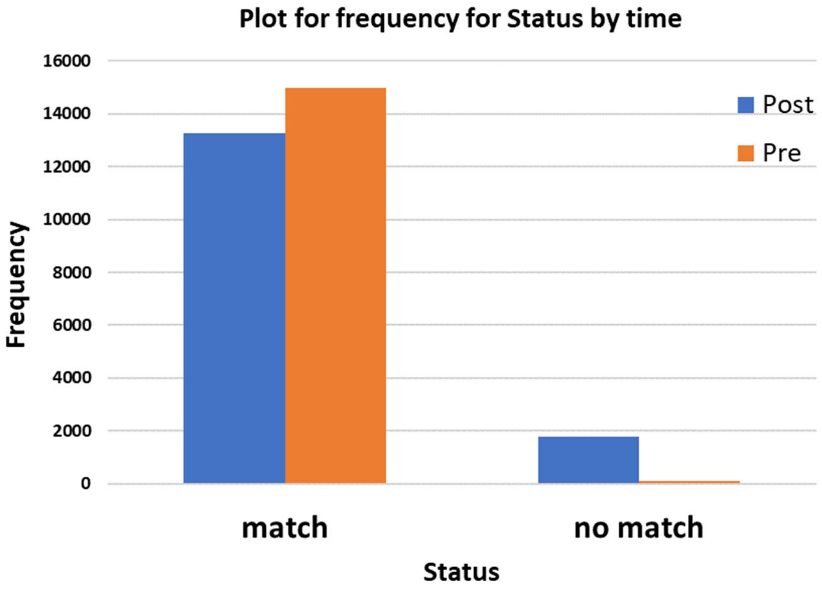

B) Plot for match vs. no match assessments for individual osseous structures

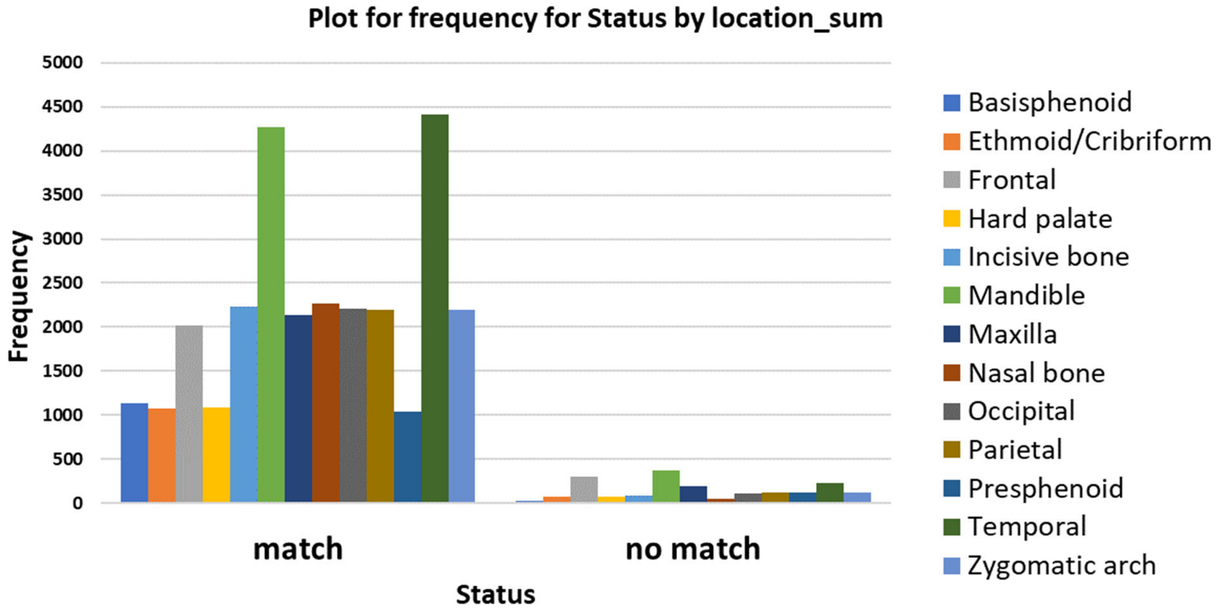

C) Plot for match vs. no match assessments for anatomic regions

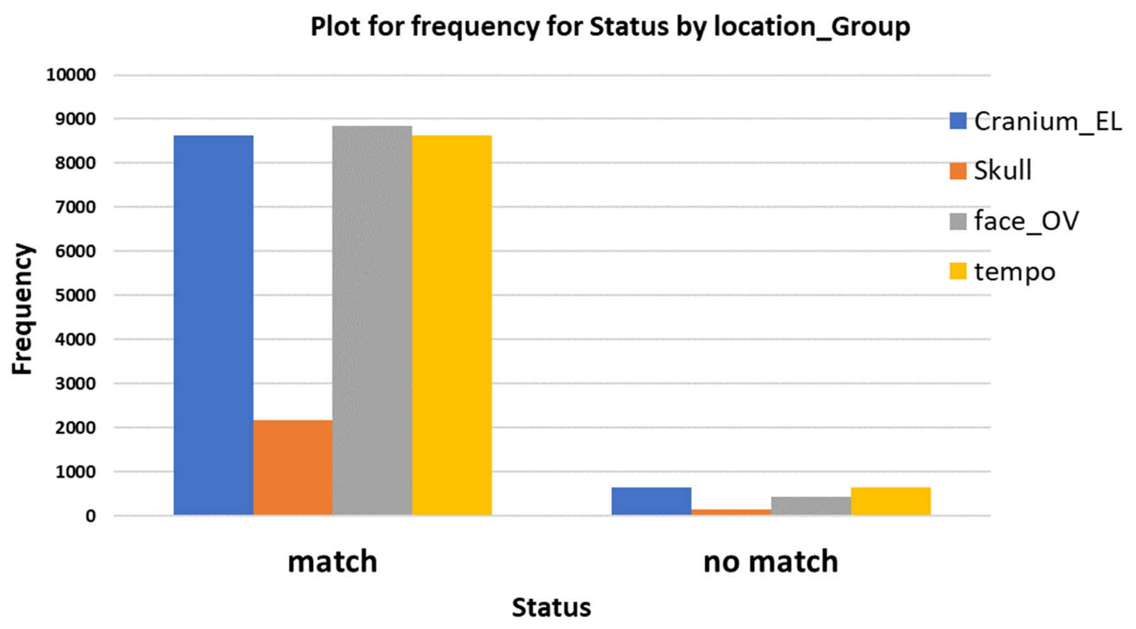

D) Plot for match vs. no match assessments for MRI sequences

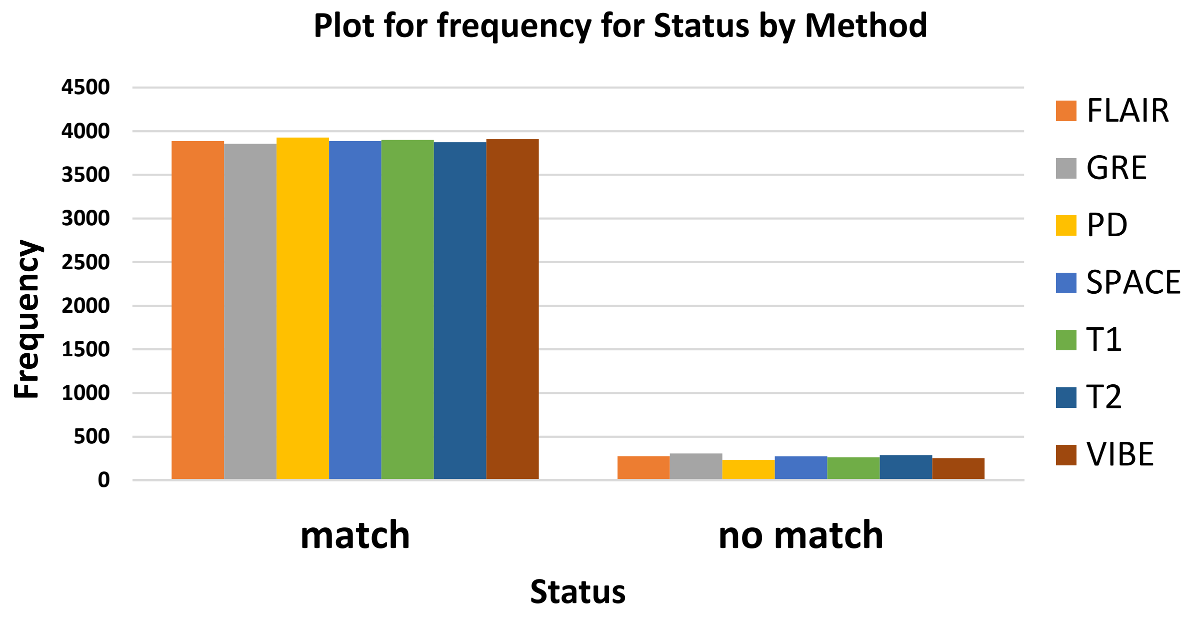

Supplement: Supplementary file 1 [file Data_Sheet_1.PDF]
